# Fast Dynamical Coupling Enhances Frequency Adaptation of Oscillators for Robotic Locomotion Control

**DOI:** 10.3389/fnbot.2017.00014

**Published:** 2017-03-21

**Authors:** Timo Nachstedt, Christian Tetzlaff, Poramate Manoonpong

**Affiliations:** ^1^Third Institute of Physics, Universität GöttingenGöttingen, Germany; ^2^Bernstein Center for Computational NeuroscienceGöttingen, Germany; ^3^Max Planck Institute for Dynamics and Self-OrganizationGöttingen, Germany; ^4^Embodied AI and Neurorobotics Lab, Centre for BioRobotics, The Mærsk Mc-Kinney Møller Institute, University of Southern DenmarkOdense, Denmark

**Keywords:** adaptive frequency oscillator, central pattern generator, neural networks, resonance tuning, locomotion control

## Abstract

Rhythmic neural signals serve as basis of many brain processes, in particular of locomotion control and generation of rhythmic movements. It has been found that specific neural circuits, named central pattern generators (CPGs), are able to autonomously produce such rhythmic activities. In order to tune, shape and coordinate the produced rhythmic activity, CPGs require sensory feedback, i.e., external signals. Nonlinear oscillators are a standard model of CPGs and are used in various robotic applications. A special class of nonlinear oscillators are adaptive frequency oscillators (AFOs). AFOs are able to adapt their frequency toward the frequency of an external periodic signal and to keep this learned frequency once the external signal vanishes. AFOs have been successfully used, for instance, for resonant tuning of robotic locomotion control. However, the choice of parameters for a standard AFO is characterized by a trade-off between the speed of the adaptation and its precision and, additionally, is strongly dependent on the range of frequencies the AFO is confronted with. As a result, AFOs are typically tuned such that they require a comparably long time for their adaptation. To overcome the problem, here, we improve the standard AFO by introducing a novel adaptation mechanism based on dynamical coupling strengths. The dynamical adaptation mechanism enhances both the speed and precision of the frequency adaptation. In contrast to standard AFOs, in this system, the interplay of dynamics on short and long time scales enables fast as well as precise adaptation of the oscillator for a wide range of frequencies. Amongst others, a very natural implementation of this mechanism is in terms of neural networks. The proposed system enables robotic applications which require fast retuning of locomotion control in order to react to environmental changes or conditions.

## 1. Introduction

Rhythmic processes are of central importance for many aspects of biological life (Winfree, 1967; Barkai and Leibler, 2000; Goldbeter et al., 2012). Examples include the cardiac rhythm, various circadian rhythms and, in particular, all forms of biological locomotion like walking, flying or swimming. The latter are controlled by specific neural circuits, so called central pattern generators (CPGs) (Hooper, 2001; Marder and Bucher, 2001). Theoretical models of CPGs range from detailed biophysical models (Hellgren and Grillner, 1992) to pure mathematical oscillators (Matsuoka, 1985). In general, CPGs can be described as nonlinear oscillators which have been used in numerous applications for different variants of robotic control problems (Nakamura et al., 2007; Ijspeert, 2008; Pinto et al., 2012; Nassour et al., 2014; Santos et al., 2017). For instance, compared to purely reflexive control schemes (Foth and Bässler, 1985; Cruse et al., 1995), oscillator-controlled robots enable more stable and robust locomotion (Kimura et al., 2001; Righetti and Ijspeert, 2008).

CPGs do not require any external input or feedback to produce basic rhythmic activity. However, they still require feedback signals to adapt and tune their produced activity, for instance its frequency. For the theoretical concept of nonlinear oscillators, a universal mechanism to adapt the intrinsic frequency of an oscillator according to the frequency of an external periodic signal, which is coupled to the oscillator, was formulated by Righetti et al. (2006). This frequency adaptation schema is applicable to many different types of oscillators. In contrast to the well-known phenomenon of entrainment, which is a purely reactive mechanism with only transient effect on the oscillatory system (Buchli et al., 2006), the frequency adaptation schema modifies the intrinsic frequency of the system permanently. Oscillators with this schema are commonly called adaptive frequency oscillators (AFOs). Several applications of AFOs have been proposed including adaptive control of compliant robots (Righetti et al., 2009), pendulum swing-up problems (Spong, 1995; Furuta, 2003), understanding, simulation and support of human locomotion (Ronsse et al., 2011a; Tropea et al., 2015; Santos et al., 2017), mimicking of fish swimming (Wang et al., 2013), frequency analysis of an input signal (Buchli et al., 2008), and construction of limit cycles of arbitrary shape (Righetti et al., 2009). However, all of these applications suffer from significantly long adaptation times.

For a given oscillatory system, the dynamics of a standard AFO is determined by only two parameters: the strength of the coupling of the external signal to the oscillator and the learning rate of the parameter determining the intrinsic frequency of the system. Here, we show that, when choosing these two parameters, one has to make a compromise between speed and precision of the resulting adaption dynamics. Furthermore, we demonstrate that the optimal parameters for a certain balance of speed and precision strongly depend on the initial intrinsic frequency of the oscillator and on the target frequency, i.e., the frequency of the external signal. As a result, situation-specific fine-tuning of the parameters is necessary.

In contrast, we propose an extension of the standard frequency adaptation mechanism which provides both fast as well as precise adaptation for a wide range of initial intrinsic and target frequencies without the need for parameter fine tuning. In the following, we call this mechanism “Adaptation through Fast Dynamical Coupling” (AFDC). It is based on dynamically adapting the coupling strength of the external signal. If the difference between the current intrinsic frequency and the target frequency is high, the coupling strength is increased in order to accelerate the adaptation. If the difference between the current intrinsic frequency and the target frequency becomes small, the coupling strength is reduced to increase the precision of the adaptation. This process is autonomous and can be integrated into the dynamical equations of the system. Neither the current intrinsic nor the target frequency need to be explicitly available as the mechanism solely relies on signal correlations. We compare the adaptation processes obtained by regular AFOs with those obtained with the new AFDC mechanism by means of quantitative measures of speed and precision of the adaptation. We find that the AFDC mechanism clearly outperforms standard AFOs within the tested frequency interval covering two orders of magnitudes.

## 2. Results

### 2.1. Standard adaptive frequency oscillator

In very general terms, an oscillator is an autonomous dynamical system with at least one limit cycle attractor (Buchli et al., 2006). Naturally, every two-dimensional oscillatory system (*x, y*) can be expressed as a system of two equations $\dot{x}$(*t*) = *g*_*x*_(*x*(*t*), *y*(*t*), θ) and $\dot{y}$(*t*) = *g*_*y*_(*x*(*t*), *y*(*t*), θ) where the functions *g*_*x*_ and *g*_*y*_ define the dynamics of the system. We require that these two functions do not only depend on the state variables *x* and *y* but also explicitly on a variable θ which determines the intrinsic oscillation frequency *f* of the system. The function *f*(θ) may be of an arbitrary shape and in many cases is not explicitly known. We only assume it to be monotonic. The system can be transformed into an adaptive frequency oscillator (AFO) by coupling it to an external signal *F*(*t*):

(1) $$\begin{array}{l} \dot{x}(t)=g_{x}(x(t),y(t),\theta (t))+\epsilon F(t)\\ \dot{y}(t)=g_{y}(x(t),y(t),\theta (t)). \end{array}$$

**Figure 1:**
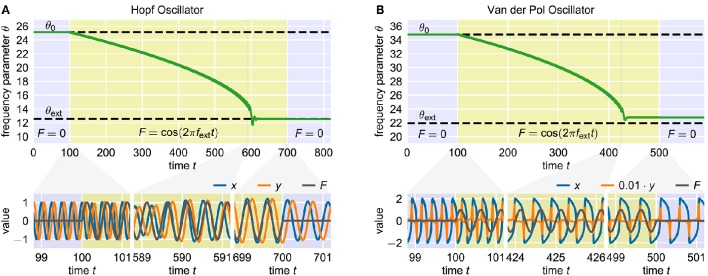
**Adaptation of two standard adaptive frequency oscillators**. The upper panels show the time course of the frequency determining parameter θ. The time during which the external signal is applied to the system is indicated by the yellow shaded area. The dashed horizontal lines indicate the values θ_0_ and θ_ext_ corresponding to the initial intrinsic frequency *f*_0_ and the target frequency *f*_ext_ of the external signal, respectively. The panels below show the oscillating state variables *x* and *y* and the external signal *F* at different short time windows during the adaptation process. In both cases, the initial intrinsic frequency of the oscillator is *f*_0_ = 4.0 and the external signal is a sine wave with unit amplitude and frequency *f*_ext_ = 2.0. **(A)** Adaptive frequency Hopf oscillator with μ = 1.0 and ϵ = η = 1.0 (see Methods). The initial value of the parameter θ is given by θ_0_ = 2π*f*_0_ ≈ 25.1. Accordingly, the value corresponding to the frequency of the external signal is θ_ext_ = 2π*f*_ext_ ≈ 12.6. The external signal is applied for 100 ≤ *t* < 700. **(B)** Adaptive frequency Van der Pol oscillator with μ = 100.0 and ϵ = η = 0.7 (see Methods). The values of the parameter θ corresponding to *f*_0_ and *f*_ext_ are θ_0_ ≈ 34.8 and θ_ext_ ≈ 22.0 (see Methods). The external signal is applied for 100 ≤ *t* < 500.

Here, ϵ denotes the coupling strength. Furthermore, additional dynamics of the θ-variable are introduced (Righetti et al., 2006):

(2) $$\dot{\theta }(t)=\pm \eta F(t)\frac{y(t)}{\sqrt{x{\left(t\right)}^{2}+y{\left(t\right)}^{2}}}.$$

with a learning rate η. The sign on the right-hand side depends on the direction of oscillation of the actual oscillatory system in the phase space. Note that in the original publication (Righetti et al., 2006), always η = ϵ is chosen as it emerges naturally when deriving the adaptation rule from analyzing the effect of the periodic external signal *F* on the phase velocity of the oscillator (Righetti et al., 2006). Apart from this, however, there is no a priori reason why this choice should provide optimal adaptation results. It has been shown that, using this rule, a wide range of oscillators can adapt their intrinsic frequencies to the frequency of basically any external periodic signal *F*(*t*). In this contribution, we consider the Hopf oscillator (Figure 1A), which possesses a harmonic limit cycle, and the Van der Pol oscillator (Van der Pol, 1920) (Figure 1B), which, depending on the choice of parameters, exhibits highly non-harmonic oscillations.

For analyzing a given adaptation process, we start with an oscillator with an initial frequency variable θ_0_ corresponding to an initial intrinsic frequency *f*_0_ = *f*(θ_0_). Here, the function *f*(θ) is not explicitly known but can be numerically approximated. We denote the target frequency, i.e., the frequency of the external signal, by *f*_ext_. Furthermore, we define the target value θ_ext_ as the value of θ such that *f*_ext_ = *f*(θ_ext_) for the given oscillator. The frequency variable θ is not modified by the adaptation rule (Equation 2) as long as the external signal *F* is zero (*t* < 100 in Figure 1A). After the onset of the external signal, θ is slowly adapted toward the target value θ_ext_ (100 < *t* < 700 in Figure 1A). The adaptation rate increases as θ gets closer to θ_ext_. The final adaptation phase is typically characterized by a small θ-overshoot before it converges toward a quasi-constant state with only small periodic fluctuations (600 < *t* < 700 in Figure 1A). Now, when removing the external signal, i.e., setting *F* = 0, the oscillator maintains oscillations at the adapted frequency (*t* > 700 in Figure 1A). Note that it is not guaranteed that the finally reached value of θ corresponds exactly to θ_ext_. In contrast, in some cases significant deviations can be observed (Figure 1B). As it turns out, reducing this deviation is only possible when accepting longer adaptation times.

#### 2.1.1. Speed vs. precision trade-off

**Figure 2:**
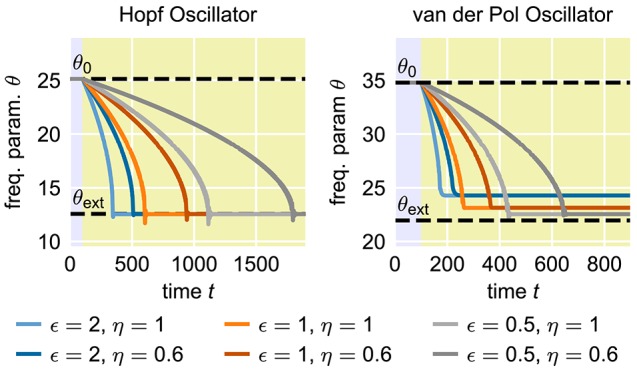
**Influence of the coupling strength ϵ and the learning rate η on the speed of adaptation of standard adaptive frequency oscillators**. The yellow shaded area indicates the time during which the external signal is applied. In all cases, the initial intrinsic frequency of the oscillator is *f*_0_ = 4.0 and the frequency of the external unit sine-wave signal is *f*_ext_ = 2.0. For the adaptive Hopf oscillator, we choose μ = 1.0. For the adaptive van-der-Pol oscillator, we choose μ = 100.0 (see Methods).

**Figure 3:**
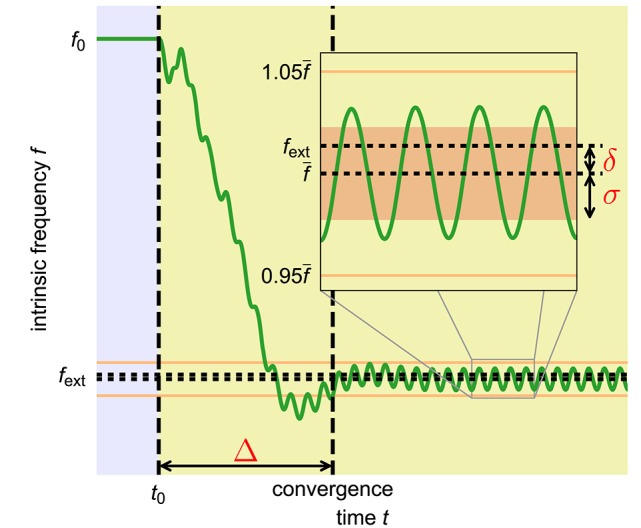
**Quantitative measures to capture the quality of an adaptation process**. Shown is the time course of the intrinsic frequency of an adaptive frequency oscillator during the adaptation to an external periodic signal with high coupling constant ϵ and high learning rate η. The yellow shaded area indicates the time during which the external signal is applied. The inset shows a close up of the data in the indicated area. We introduce three measures to quantify the quality of a given frequency adaption process. The convergence time Δ is the time interval between the onset of the external signal at time *t*_0_ and the last deviation of the intrinsic frequency of the oscillator of more than 5% (orange horizontal lines) from the finally reached average intrinsic frequency $\overset{-}{f}$. The frequency offset δ measures the difference between the final average intrinsic frequency $\overset{-}{f}$ and the target frequency of the external signal *f*_ext_. In order to also capture the periodic fluctuations of the intrinsic frequency from the average value $\overset{-}{f}$, we additionally introduce the final frequency fluctuation σ given by the standard deviation of the oscillations of the intrinsic frequency *f* in the finally reached state (area shaded in light red in the inset). The shown time course of the intrinsic frequency is taken from an adaptive frequency Hopf oscillator with μ = 1.0, ϵ = 5.0, η = 5.0, and *f*_0_ = 2.0 adapting to an external unit sine-wave signal with frequency *f*_ext_ = 1.0.

In many applications, for instance in robotic systems, it is usually desired to have systems that are able to adapt to new situations or circumstances quickly. In contrast, AFOs with the usual choice of parameters require many periods of oscillations to complete a given adaptation process. The convergence time of the adaptation process, i.e., the time between the onset of the external signal and the quasi-convergence of the frequency parameter θ of the oscillator, can be adjusted by manipulating the coupling strength ϵ in Equation (1) or the learning rate η in Equation (2) (Figure 2). However, increasing ϵ or η does not only increase the speed of the frequency adaptation but also increases the general influence of the external signal on the oscillatory system. As a result, the dynamics of the parameter θ, once it has converged to a quasi-stable state, is affected as well (Figure 3). On the one hand, high learning rates η lead to increased fluctuations of the parameter θ in the finally reached state. On the other hand, higher values of ϵ result in a higher offset of the finally reached mean value $\overset{-}{\theta }$ from the value θ_ext_. Therefore, shorter convergence times in the standard AFO systems go hand in hand with a loss of precision. Naturally, this trade-off complicates real-world applications of the mechanism.

#### 2.1.2. Quantitative adaptation quality measures

In order to quantitatively capture the trade-off between speed and precision, we introduce three measures characterizing the quality of a given adaptation process (Figure 3). As already discussed, in many applications fast adaptation is desired. This is captured by the convergence time Δ which measures the time interval between the onset of the external signal and the last deviation of the intrinsic frequency *f* of the system (determined by θ) of more than 5% (10% for the Van der Pol oscillator) from the finally reached mean value $\overset{-}{f}$. The precision of the adaptation, in turn, is reflected by two measures. First, the intrinsic frequency to which the system converges should be as close as possible to the frequency of the external signal. This is measured by the frequency offset δ which is given by the offset of the finally reached mean value of the intrinsic frequency from the frequency of the external signal. Second, the fluctuations of the intrinsic frequency around its mean value should be low as otherwise the value of the intrinsic frequency when switching off the external signal depends on the exact point of time of this event. The magnitude of these fluctuations is measured by σ which equals the standard deviation of the intrinsic frequency *f* in the converged state.

To allow interpretation of these measures independently from the chosen internal and external frequencies, we introduce relative measures scaled by the frequency *f*_ext_ or the cycle duration $f_{\text{ext}}^{-1}$ of the external signal, respectively: $\tilde{\Delta }=\Delta /f_{\text{ext}}^{-1}$, $\tilde{\delta }=\delta /f_{\text{ext}}$ and $\tilde{\sigma }=\sigma /f_{\text{ext}}$. In addition, we define a quality index *Q* combining these three relative measures into a single scalar value:

(3) $$Q=\text{max}\left(1-\frac{\tilde{\Delta }}{{\tilde{\Delta }}_{\text{max}}}-\frac{\left|\tilde{\delta }\right|}{{\tilde{\delta }}_{\text{max}}}-\frac{\tilde{\sigma }}{{\tilde{\sigma }}_{\text{max}}},0\right).$$

Here, ${\tilde{\Delta }}_{\text{max}}$, ${\tilde{\delta }}_{\text{max}}$, and ${\tilde{\sigma }}_{\text{max}}$ are the maximum values of the respective measures which we allow for a reasonably good adaptation process. Accordingly, a *Q* value close to 1 corresponds to a very fast as well as very precise adaptation process. A value of *Q* = 0, in contrast, indicates that $\tilde{\Delta }>{\tilde{\Delta }}_{\text{max}}$, $\tilde{\delta }>{\tilde{\delta }}_{\text{max}}$, $\tilde{\sigma }>{\tilde{\sigma }}_{\text{max}}$ or the weighted sum (Equation 3) of the individual measures is larger than 1. In the following, if not stated otherwise, we use ${\tilde{\Delta }}_{\text{max}}=100$, ${\tilde{\delta }}_{\text{max}}=0.05$ and ${\tilde{\sigma }}_{\text{max}}=0.05$.

#### 2.1.3. Finding optimal parameters

**Figure 4:**
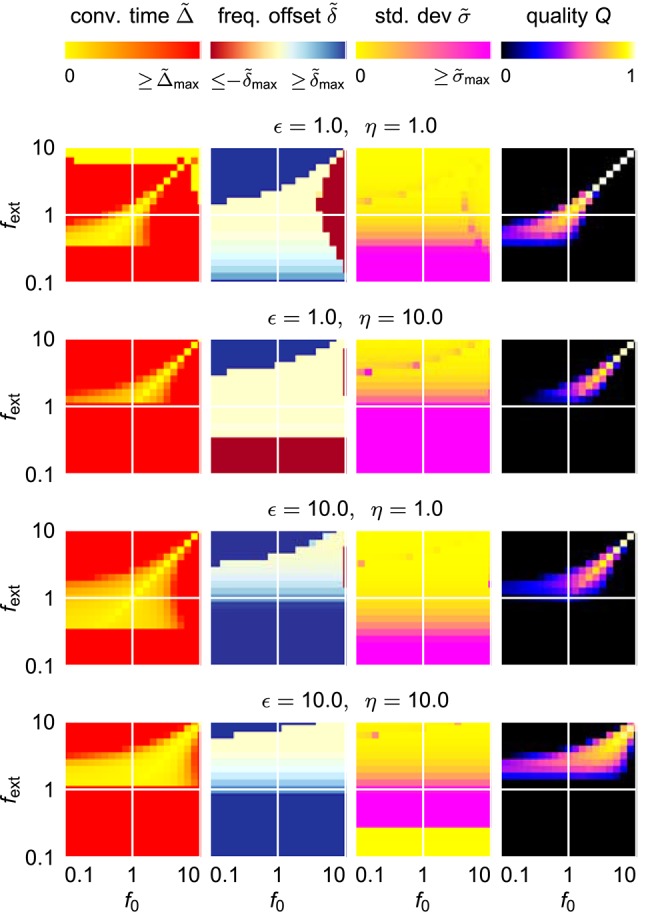
**Adaptation quality measures of the adaptive frequency Hopf oscillator in the (***f***_**0**_, ***f***_**ext**_) frequency space for different values of the coupling strength ϵ and the learning rate η**. For every given (ϵ, η)-parameter pair, from left to right, the relative convergence time $\tilde{\Delta }$, the relative final frequency offset $\tilde{\delta }$, the final relative frequency fluctuation $\tilde{\sigma }$, and the combined quality measure *Q* are shown in the plane spanned by the initial intrinsic frequency *f*_0_ and the frequency of the external unit sine-wave signal *f*_ext_. As the convergence time is defined as the time difference between the onset of the external signal and the last point of time of more than 5% deviation of the intrinsic frequency from the final average, for high values of $\tilde{\sigma }$, the convergence time cannot be reasonably determined, i.e., takes very high values. For the same reason, even on the diagonal *f*_0_ = *f*_ext_, high convergence times are measured for low values of *f*_ext_.

For an easy application of an adaptive oscillator in a given setup, no fine tuning of the system parameters for the specific application context should be necessary. It is therefore necessary to find a system which is able to adapt its intrinsic frequency to a wide range of external frequencies without the need for any parameter adaptation other than the one of the frequency determining parameter θ. It turns out, however, that already for the comparable simple case of the harmonic Hopf oscillator, the range of frequencies for which a given set of parameters allows fast as well as precise adaptations is very limited (Figure 4). Higher values of ϵ and η increase the intervals of initial intrinsic frequencies *f*_0_ and external frequencies *f*_ext_ for which fast adaptation is achieved (left column in Figure 4). In contrast, small frequency offsets $\tilde{\delta }$ are achieved only for small values of the coupling strength ϵ (second column in Figure 4) and small values of the learning rate η enable small fluctuations as measured by σ (third column in Figure 4). The compilation of these observations is reflected by only small intervals of initial intrinsic and external frequencies for which the quality index *Q* attains non-zero values (right column in Figure 4).

**Figure 5:**
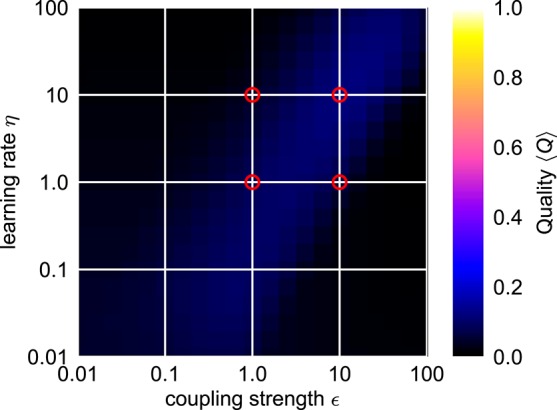
**Average combined quality measure 〈***Q***〉 for different parameter values of the frequency adaptive Hopf oscillator**. For every parameter pair of coupling strength ϵ and learning rate η, the average adaption quality measure 〈*Q*〉 over the logarithmically sampled space of initial intrinsic frequencies *f*_0_ and frequencies of the external signal *f*_ext_ is shown (0.1 < *f*_0_, *f*_ext_ < 10). The red circles indicate the four cases shown in Figure 4. In each case, the external signal is a sine-wave with unit amplitude.

Trying to find parameters that allow fast and precise adaptation for a range of initial intrinsic and external target frequencies spanning two orders of magnitudes reveals that actually no ϵ-η-combination allows for an average adaptation quality index 〈*Q*〉 higher than approximately 0.12 (Figure 5). We conclude that a standard AFO with a fixed set of parameters is not capable to provide fast as well as precise adaptation over a wide range of frequencies.

### 2.2. Fast dynamical coupling mechanism

As discussed, no fixed value pair for the coupling strength ϵ and the learning rate η suffices for fast and precise adaptation over a wider range of initial intrinsic and external target frequencies. In order to obtain a system without the requirement for application-specific fine-tuning, the down- or up-scaling of coupling strength and learning rate has to be accomplished in a self-organized manner. Here, we propose such a system. Instead of coupling the external signal *F*(*t*) directly to the oscillator, we now use a filtered signal *P*(*t*):

(4) $$\begin{array}{l} \dot{x}(t)=f_{x}(x(t),y(t),\theta (t))+P(t)\\ \dot{y}(t)=f_{y}(x(t),y(t),\theta (t)). \end{array}$$

Accordingly, also the adaptation of θ is based on *P*(*t*):

(5) $$\dot{\theta }(t)=\pm \eta P(t)\frac{y(t)}{\sqrt{x{\left(t\right)}^{2}+y{\left(t\right)}^{2}}}.$$

*P*(*t*) is given by a weighted difference of the external signal *F*(*t*) and the oscillator variable *x*(*t*):

(6) P(t)=ϵ(t)F(t)−β(t)x(t)

with the adaptive coupling strengths ϵ(*t*) and β(*t*). Following the discussion of the quality measures introduced before, for an optimal adaptation process, the dynamics of ϵ(*t*) and β(*t*) has to fulfill two requirements: as long as the difference between the intrinsic frequency *f* and the target frequency *f*_ext_ of the external signal is high, *P*(*t*) should basically be an amplified version of *F*(*t*) in order to accelerate the adaptation process. In contrast, when *f* is already close to *f*_ext_, *P*(*t*) is supposed to attain values close to zero such as to reduce the influence of the external signal to a minimum. Both of these requirements can be fulfilled by adapting β(*t*) and ϵ(*t*) according to a combination of correlation-based growth and a passive decay toward a low resting value. For β(*t*), we propose the following dynamics:

(7) $$\tau \dot{\beta }(t)=\beta_{0}-\beta (t)+\kappa P(t)x(t)$$

with time constant τ and correlation learning rate κ. The value of β scales the subtraction of the system variable *x* from the external signal *F*(*t*) in Equation (6). The product of *P* and *x* (averaged over time) is large if the difference between the intrinsic frequency *f* and the external target frequency *f*_ext_ is low. At this stage, the influence of the external signal on the oscillator should be reduced, i.e., the amplitude of *P* should be decreased, as done by increasing β. The proposed dynamics for ϵ(*t*) are very similar:

(8) $$\tau \dot{\epsilon }(t)=\epsilon_{0}-\epsilon (t)+\kappa F(t)P(t).$$

The value of ϵ scales the influence of the external signal *F* on the filtered signal *P* (Equation 6). If the averaged product of *F* and *P* is large, this implies that the subtraction of *x* in Equation (6) cannot cancel the addition of *F*, i.e., the internal frequency of the oscillator is different from the target frequency of the external signal. Thus, an increase of ϵ is desired to increase the influence of the signal on the system and to herewith increase the adaptation speed. However, as for β(*t*) ≈ 0, the last term of Equation (8) can be approximated by κ*F*(*t*)^2^ϵ(*t*), without adaptation of β(*t*), the value of ϵ(*t*) would not return to ϵ_0_ as long as the external signal is present and therefore would not allow precise adaptation. Only the interplay of the dynamics of ϵ(*t*), which detects the onset of an external signal with a frequency different from the intrinsic frequency of the oscillator, and of β(*t*), which detects when the adaptation is nearly completed, allows fast as well as precise adaptation. In the following, we call the described mechanism “Adaptation through Fast Dynamical Coupling” (AFDC).

**Figure 6:**
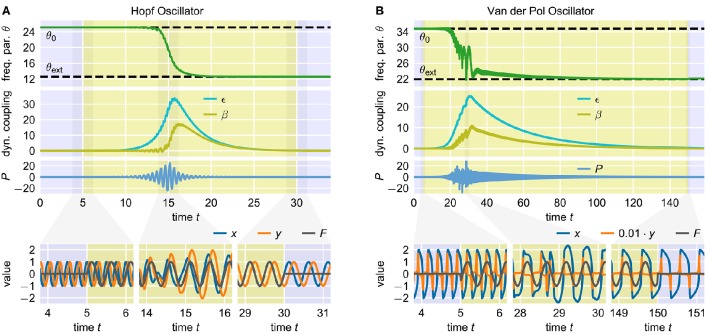
**Adaptation of two oscillators with the AFDC mechanism**. The upper-most panels show the time course of the frequency determining parameter θ. The time during which the external signal is applied to the system is indicated by the yellow shaded area. The dashed horizontal lines indicate the initial value θ_0_ and the value θ_ext_ corresponding to the exact frequency of the external signal. The second panels from the top show the time course of the adaptive coupling strengths β and ϵ. The third panels from the top show the time course of the filtered external signal *P*. The panels on the bottom show the oscillating state variables *x* and *y* and the external signal *F* at different short time windows during the adaptation process. In both cases, the initial intrinsic frequency of the oscillator is *f*_0_ = 4.0 and the external signal is a sine wave with unit amplitude and frequency *f*_ext_ = 2.0. **(A)** Hopf oscillator with AFDC mechanism with μ = 1.0, η = 0.5, κ = 5.0, τ = 2.0, β_0_ = 0.0 and ϵ_0_ = 0.01. The initial value of the parameter θ is given by θ_0_ = 2π*f*_0_ ≈ 25.1, the value corresponding to the frequency of the external signal is θ_ext_ = 2π*f*_ext_ ≈ 12.6. The external signal is applied for 5 ≤ *t* < 30. **(B)** Van der Pol oscillator with AFDC mechanism with μ = 100.0, η = 2.0, κ = 5.0, τ = 15.0, β_0_ = 0.0 and ϵ_0_ = 0.01. The values of the parameter θ corresponding to *f*_0_ and *f*_ext_ are determined to be θ_0_ ≈ 34.8 and θ_ext_ ≈ 22.0 (see Methods). The external signal is applied for 5 ≤ *t* < 150.

The process of frequency adaptation supported by the AFDC mechanism can be separated into several stages (Figure 6) as qualitatively described in the following: Before the onset of an external signal (*F* = 0), the average product of *P* and *F* is zero and the adaptive coupling constants β and ϵ converge toward their resting values $\beta_{0}/\left(1+\kappa \overset{-}{x^{2}}\right)$ and ϵ_0_. Here, $\overset{-}{x^{2}}$ is the mean over time of the squared signal *x*^2^. As soon as the external signal is applied, the average product of *P* and *F* gets positive (Equation 6). As a result of this, ϵ starts to increase (Equation 8). A higher value of ϵ, in turn, increases the average product of *P* and *F*. This establishes a positive feedback loop that leads to a fast increase of the amplitude of *P*. The high amplitude of *P* results in a large influence of the external signal on the oscillator (Equation 4) as well as in a fast adaptation of the frequency determining variable θ toward the frequency of *F* (Equation 5). As a consequence of both of these effects, the oscillator follows the external frequency implying a positive correlation between *P* and *x*. This correlation leads to an increase of β (Equation 7). Higher values of β decrease the amplitude of *P* (Equation 6) and, as such, also the average product between *P* and *x*. This is a negative feedback loop. Note that a decrease of the amplitude of *P* also reduces the average product of *P* and *F* and therefore breaks the positive feedback loop between ϵ and the average product of *P* and *F* (Equation 8) yielding a decline of both β and ϵ to their respective resting values. At this point, switching off the external signal does not significantly change the system dynamics as the influence of the external signal has already been reduced to a minimum.

In summary, the described interplay of the dynamics of the two adaptive coupling constants β and ϵ scales up the magnitude of the external signal as long as adaptation of θ is needed and reduces it once the value corresponding to the frequency of the external signal is reached.

#### 2.2.1. Adaptation quality in frequency space

**Figure 7:**
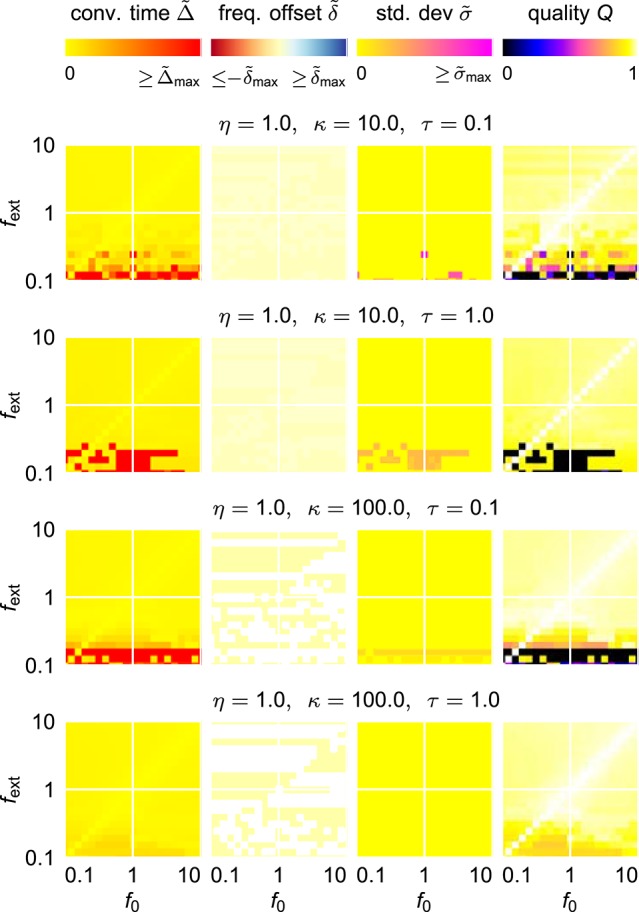
**Adaptation quality measures for the Hopf oscillator with AFDC mechanism in the ***f***_**0**_-***f***_**ext**_-frequency space for different parameter values**. For each of the given parameter tuples (η, κ, τ), from left to right, the relative convergence time $\tilde{\Delta }$, the relative final frequency offset $\tilde{\delta }$, the final relative frequency fluctuation $\tilde{\sigma }$, and the combined quality measure *Q* for the given parameter values of η, κ, and τ are shown in the plane spanned by the initial intrinsic frequency *f*_0_ and the frequency of the external unit sine-wave signal *f*_ext_.

The dynamics of the AFDC mechanism is mainly dominated by three free parameters: The time scale τ of the adaptive coupling strengths, the correlation learning rate κ and the learning rate η of the frequency determining variable θ. While, in general, an oscillator equipped with an AFDC mechanism shows more tolerance with respect to large frequency ranges, certain parameter combinations allow a faster or more precise adaptation over a larger frequency range (Figure 7). Some combinations (for instance η = 1.0, κ = 100.0, τ = 1.0) result in a very good performance, as indicated by high values of *Q*, for the complete range of initial intrinsic frequencies *f*_0_ and frequencies *f*_ext_ of the external signal analyzed here.

**Figure 8:**
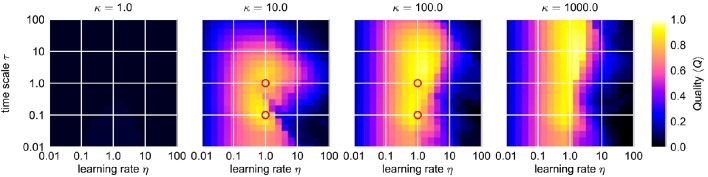
**Average combined quality measure 〈***Q***〉 for different values of κ in the ϵ-η-parameter space of the Hopf oscillator with AFDC mechanism**. For every parameter triple of coupling strength ϵ, frequency learning rate η and correlation learning rate κ, the average adaption quality measure 〈*Q*〉 over the logarithmically sampled space of initial intrinsic frequencies *f*_0_ and frequencies of the external signal *f*_ext_ is shown (0.1 < *f*_0_, *f*_ext_ < 10). In each case, the external signal is a sine-wave with unit amplitude. The red circles indicate the four cases shown in Figure 7. Comparing these results to the ones obtained for the standard AFO in Figure 5, the AFDC mechanism provides significant higher quality values indicating versatility with respect to different initial intrinsic and external target frequencies.

**Figure 9:**
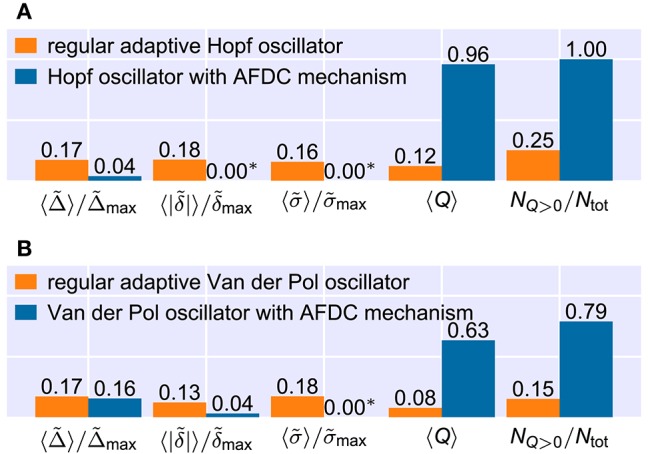
**Comparison of the frequency space averaged adaptation quality measures for the best found configurations of the regular adaptive oscillators and the respective oscillators with AFDC mechanism**. Note that the averages of the relative convergence time $\tilde{\Delta }$, the final frequency offset $\left|\tilde{\delta }\right|$ and the relative final frequency fluctuation $\tilde{\sigma }$ include only values from (*f*_0_, *f*_ext_)-frequency pairs in which the combined quality measure *Q* has a nonzero value. The ratio of the number *N*_*Q*>0_ of (*f*_0_, *f*_ext_)-pairs for which the quality *Q* has a nonzero value and the total number *N*_tot_ of frequency pairs is shown on the very right. All numbers are rounded. See methods for the used parameter values. **(A)** For the Hopf oscillator, all parameters are identical to the ones used in Figures 7, 8. **(B)** For the Van der Pol oscillator, we adapt the maximal allowed values of the quality measures. We use ${\tilde{\Delta }}_{\text{max}}=200$, ${\tilde{\delta }}_{\text{max}}=0.10$ and ${\tilde{\sigma }}_{\text{max}}=0.05$. In addition, we calculate $\tilde{\delta }$ and $\tilde{\sigma }$ directly from the frequency determining variable θ and consider the last deviation of θ of more than 10% from the finally reached mean value $\overset{-}{\theta }$ to determine the adaptation time Δ. (*) Values shown as 0.00 are too small to be resolved in the figure. For the Hopf oscillator with AFDC mechanism, we find $\langle \tilde{|}\delta |\rangle /{\tilde{\delta }}_{\text{max}}\approx 5.3\cdot 10^{-7}$ and $\langle \tilde{\sigma }\rangle /{\tilde{\sigma }}_{\text{max}}\approx 8.5\cdot 10^{-8}$. For the Van der Pol oscillator with AFDC mechanism, the average normalized final frequency fluctuation is $\langle \tilde{\sigma }\rangle /{\tilde{\sigma }}_{\text{max}}\approx 2.5\cdot 10^{-3}$.

This is also reflected by the frequency space averaged quality 〈*Q*〉 (Figure 8). For a sufficiently high κ (κ ≳ 3), parameters ϵ and η can be found with an average quality value close to the theoretical maximum of 1 corresponding to very fast adaptation without significant frequency offset or frequency oscillations in the finally reached state (Figure 8). A comparison of the performance of the best found configuration of the regular adaptive Hopf oscillator with the performance of the best found configuration of the Hopf oscillator with AFDC mechanism shows that the AFDC mechanism outperforms the regular AFO mechanism in terms of all quality measures (Figure 9A). The same holds true for the comparison of the regular adaptive Van der Pol oscillator with the respective AFDC implementation (Figure 9B). In contrast to a regular AFO, the AFDC mechanism manages to provide fast and precise frequency adaptation over a wide frequency range with a fixed set of parameters.

Note that the values of the additional parameters ϵ_0_ and β_0_ do not significantly influence the dynamics of the mechanism as long as they are chosen reasonably low.

#### 2.2.2. Neural implementation

The AFDC mechanism relies on dynamically adapting the coupling strengths ϵ and β. In terms of signal flow, ϵ can be interpreted as a feedforward coupling from the external signal to the filtered signal *P*. The value of β, in turn, determines the strength of feedback coupling from the oscillator back to *P*. A standard way to implement this kind of signal flow between different entities is in terms of artificial neural networks. Neural networks are composed of multiple comparably simple computational units, the neurons, which project signals to each other via so-called synapses. Every synapse is characterized by a scalar value, the synaptic weight, which determines the efficacy of the synaptic signal transmission.

There exist neuron models on many different levels of abstraction, ranging from simple binary units to complex biophysical plausible spiking models. Here, we restrict ourselves to a very basic model of point-like neurons described by time-discrete dynamics. It has been shown that already a fully connected network of only two of these very simple neurons suffices to autonomously produce oscillatory signals (Pasemann et al., 2003). In every time step, each neuron sums up the incoming outputs from other neurons as well as from itself weighted by the respective synaptic weights. This sum is transformed into the new neural output by means of a sigmoidal transfer function. The weight matrix of this two neuron network is given by a scaled rotational matrix for a rotation angle φ. The value of φ monotonically controls the frequency of the obtained oscillatory signal of this so-called SO(2)-oscillator.

**Figure 10:**
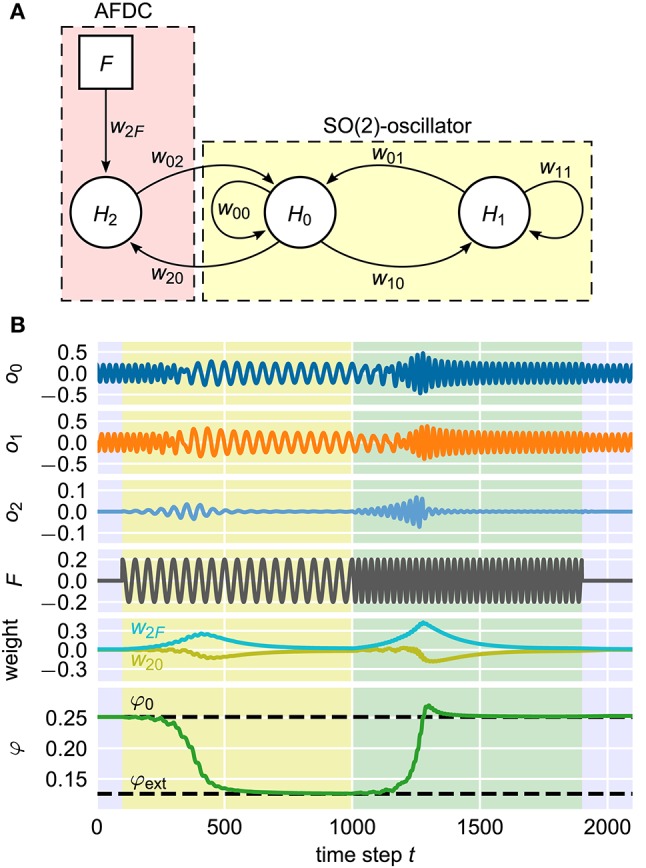
**Neural implementation of the AFDC mechanism**. **(A)** The neurons *H*_0_ and *H*_1_ are fully connected by the synapses *w*_00_, *w*_01_, *w*_10_, and *w*_11_ and form a neural SO(2)-oscillator. The neuron *H*_2_ calculates the signal *P* which is the weighted difference between the external signal *F* and the activity value of *H*_0_. Accordingly, the weight *w*_2*F*_ corresponds to the coupling strength ϵ and the weight *w*_20_ represents the variable β of the AFDC mechanism. The weight *w*_02_ can either be fixed at a positive value or adapted with similar dynamics as *w*_20_ and *w*_2*F*_. **(B)** Example adaptation of the neural oscillator. It is initialized with an intrinsic frequency of *f*_0_ = 0.04 corresponding to a value of φ_0_ = 0.25 of the internal frequency determining variable. At time step *t* = 100, an external signal with a frequency of *f*_ext_ = 0.02 is applied until time step *t* = 1, 000 (yellow shaded area). For 1, 000 < *t* < 1, 900, the frequency of the external signal is changed to *f*_ext_ = 0.04 (green shaded area). For *t* ≥ 1, 900, there is no external signal. Shown from top to bottom are the activities *o*_*i*_ of the neurons *H*_*i*_ (*i* ∈ {1, 2, 3}), the external signal *F*, the synaptic weights *w*_20_ and *w*_2*F*_ and the frequency determining variable φ of the SO(2)-oscillator.

As already shown earlier (Nachstedt et al., 2012), a neural SO(2)-oscillator with neurons *H*_0_ and *H*_1_ can be extended by an AFDC mechanism by introducing an additional neuron *H*_2_ into the system (Figure 10A). Now, this neural implementation can be understood as a special implementation of the general AFDC mechanism. The additional neuron *H*_2_ is used to calculate the filtered external signal *P* by receiving synapses from both the external input *F* and the output of neuron *H*_0_. The latter takes the role of the variable *x* of the general oscillators discussed above. The synaptic weight *w*_2*F*_ of the synapse from the external signal *F* to the additional neuron *H*_2_ implements the dynamics of the ϵ coupling. The weight *w*_20_ of the synapse from the oscillator neuron *H*_0_ to the neuron *H*_2_ takes the role of β. Adapting the synaptic weights according to Equations (7) and (8) effectively introduces synaptic plasticity into the system (Abbott and Nelson, 2000). In contrast to earlier publications (Nachstedt et al., 2012), here, the weight *w*_02_ of the synapse feeding the filtered signal *P* into the oscillator is simply kept constant.

The adaptation of the intrinsic oscillation frequency by modifying the parameter φ and hereby the synaptic weights of the neural SO(2)-oscillator is a long-lasting change. The discussed plasticity of the synaptic weights *w*_20_ and *w*_2*F*_, in contrast, has a transient character. The combination of these two different kinds of dynamics results in a fast and precise adaptive neural oscillator (Figure 10B) (Nachstedt et al., 2012). This shows that the AFDC mechanism can be easily integrated into existing neural control schemes, for instance, in robotic applications. In addition, the successful implementation of the AFDC mechanism in a time-discrete system shows that the concept can be generalized to this class of dynamical systems.

#### 2.2.3. Closed-loop locomotion control

In addition to the open-loop scenarios studied so far, the AFDC mechanism also allows to apply adaptive oscillators in closed-loop scenarios where fast adaptation toward a specific frequency is required. A classical problem of robotic locomotion control is the task to find the optimal frequency to drive the legs of a walking machine. For animals, it has been found that the frequency during locomotion is tightly related to the resonant frequency of the free swinging leg (Holt et al., 1990). This way, animals are able to maintain energy efficiency during locomotion (Ahlborn and Blake, 2002). Furthermore, it has been proposed that animals actively modify the resonant frequency of their legs in order to optimize for different walking speeds (Ahlborn and Blake, 2002).

**Figure 11:**
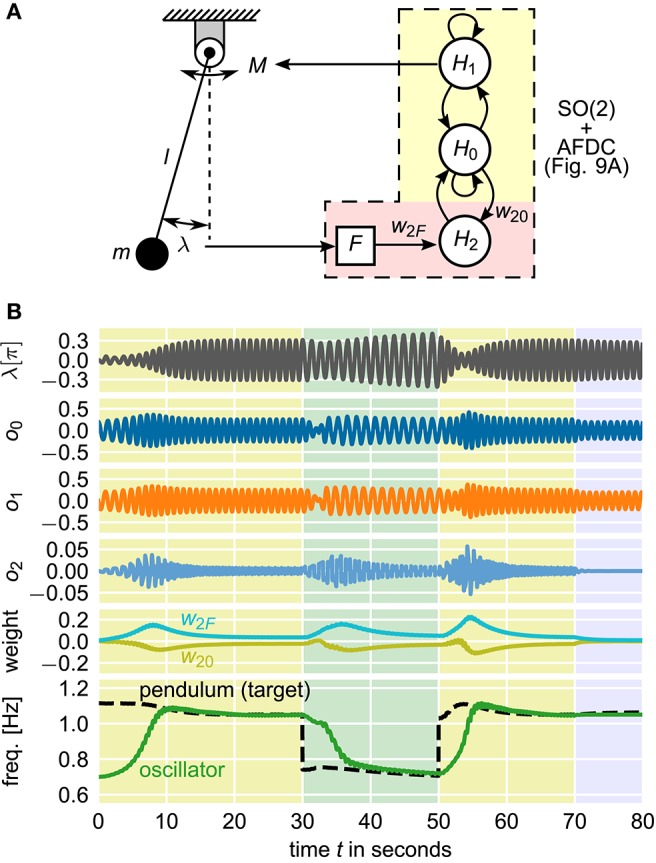
**Closed-loop pendulum control using a neural SO(2)-oscillator with AFDC mechanism**. Energy-efficient control is realized if the pendulum is driven at its resonant frequency. **(A)** The output *o*_1_ of neuron *H*_1_ controls the torque *M* driving the pendulum with length *l* and mass *m*. The current angular displacement λ is converted into the external signal *F* which is fed back to the adaptive oscillator. The neural network is updated with a frequency of 25*Hz*. **(B)** Simulation of the system with varying pendulum length *l*. The initial length of the pendulum is *l*_0_ = 0.2m. At *t* = 30 s, the length is changed to *l*_1_ = 0.4m. At *t* = 50 s, the original length *l*_0_ is restored. At *t* = 70 s, the feedback connection from the pendulum to the oscillator is cut to demonstrate that the oscillator has indeed learned the correct frequency to drive the pendulum. Shown are the current angular displacement λ of the pendulum, the outputs *o*_0_, *o*_1_, and *o*_2_ of the three neurons, the synaptic weights *w*_2*F*_ and *w*_20_ of the plastic synapses of the AFDC mechanism, and the intrinsic frequency of the oscillator and the resonant frequency of the undamped and undriven pendulum (target frequency for the oscillator). The resonant frequency of the pendulum does not only depend on the current physical properties of the pendulum but also on the current amplitude of its oscillations.

Given that CPGs control locomotion, adaptation of CPGs toward a system's resonant frequency to optimize locomotion has been repeatedly investigated and modeled (Verdaasdonk et al., 2006, 2009). A simplistic model of this control problem is given by a mathematical pendulum which is driven by a torque signal according to the output of an oscillator (Nachstedt et al., 2012). The most energy-efficient control is achieved if the pendulum is driven at its resonant frequency determined by its physical length *l* and its mass *m* as well as the current amplitude of its oscillation. Here, a neural SO(2)-oscillator with AFDC mechanism is used to control the torque applied to the pendulum. The control loop is closed by feeding the current position of the pendulum as external signal back to the oscillator (Figure 11A).

In this closed-loop system, the current frequency of the pendulum is completely determined by the current frequency of the driving neural oscillator. The observed oscillation frequencies of the pendulum and the neural oscillator are therefore always identical. Still, it is possible to adapt the intrinsic frequency of the neural oscillator to the target frequency given by the resonant frequency of the pendulum. The information about the difference between the intrinsic frequency of the oscillator and the resonant frequency of the pendulum is encoded in the phase relation between the internal oscillation and the feedback signal received as external signal by the oscillator. In particular, driving the pendulum at its resonant frequency is characterized by a phase shift of π/2 between the applied torque and the current angular position of the pendulum. In the neural SO(2)-oscillator, the same phase shift is found between the outputs of the neurons *H*_0_ and *H*_1_. Therefore, when using the output of *H*_1_ to control the torque applied to the pendulum, at resonant frequency, the current angular position of the pendulum is exactly in phase with the output of neuron *H*_0_. This corresponds to the converged state of the AFDC mechanism. If, in turn, the oscillation frequency of the neural SO(2)-oscillator is different from the resonant frequency of the pendulum, the output of *H*_0_ and the angular position of the pendulum are not in phase. The respective phase difference encodes the information about the difference between the intrinsic frequency of the neural oscillator and the resonant frequency of the pendulum and allows the adaptation of the former into the direction of the latter.

In our simulation, we first let the neural SO(2)-oscillator with AFDC mechanism adapt its intrinsic frequency toward the pendulum's resonant frequency (0 s < *t* < 30 s in Figure 11B). We then simulate a change of the physical properties of the driven system by abruptly changing the length *l* of the pendulum. Accordingly, the neural oscillator readapts its intrinsic frequency to the new resonant frequency of the pendulum (30 s < *t* < 50 s in Figure 11B). Afterwards, we change the length *l* back to its original value. Finally, we cut the feedback connection from the pendulum to the oscillator (*t* > 70 s in Figure 11B) demonstrating that the oscillator has actually learned the proper frequency to drive the pendulum.

## 3. Discussion

Transferring key concepts of biological solutions for complex control problems to robotic applications has been proven to be a promising approach regarding the adaptivity, robustness, versatility and agility found in biological organisms (Pfeifer et al., 2007). One especially successful concept is the one of using oscillators, i.e., CPGs, to control complex locomotion. As such, the study of nonlinear oscillators, their entrainment and adaptation properties and possible applications in robotics has gained a lot of interest. The here presented AFDC mechanism overcomes the demonstrated trade-off between speed and precision inherent to regular AFOs as introduced by Righetti et al. (2006). As a result, the AFDC mechanism allows fast and precise adaptation to external signals for a wide range of frequencies with a fixed set of parameters.

Since the discovery of the AFO mechanism, various different mechanisms to improve or extend the adaptation capabilities have been proposed. Subtracting the output of an oscillator from the external signal, as also done in the AFDC mechanism, was used to decompose a signal into its Fourier components (Ronsse et al., 2011b) with the help of an array of AFOs. In order to more reliably find the basic frequency of the external signal, it was proposed to combine a single adaptive frequency oscillator with a Fourier decomposition (Petric et al., 2011). The detailed interaction between multiple AFOs has been studied in the context of networks of self-adaptive dynamical systems (Rodriguez and Hongler, 2014). As an alternative to adapting the system parameters in order to modify the frequency of an oscillator, switching between different oscillation frequencies of an oscillator operated in the chaotic regime by dynamically stabilizing different periods was demonstrated (Steingrube et al., 2010).

The main novelty of the here presented mechanism is the introduced dynamics of the adaptive coupling strengths between the external signal and the filtered signal as well as between the output of the oscillator and the filtered signal. This dynamics temporally increases the influence of the signal on the oscillator as long as it is necessary to achieve fast adaptation and decreases it once precision is needed toward the end of the adaptation process. Adaptive coupling strengths have been proposed earlier as a method to increase the synchronization in a network of phase oscillators with fixed intrinsic frequencies (Ren and Zhao, 2007). The interaction of the transient dynamics of the adaptive coupling strengths on the one hand and the permanent change of intrinsic frequency on the other hand resembles the interplay of long-term (Wood et al., 2011) and short-term (Zucker and Regehr, 2002) plasticity in biological organisms. The interplay of long-term and short-term plasticity in biological system has already been shown to be highly relevant for biological motor control, in particular for fast network reconfiguration (Nadim and Manor, 2000).

The AFDC mechanism increases the complexity of the oscillatory system by the addition of two dynamical equations. Their interplay is required to first scale up the influence of the external signal and later on reduce it again. In particular, this interplay is enabled by the weighted difference *P* of the external signal *F* and the oscillator variable *x*. The correlation of *P* and *F* determines the growth of the adaptive coupling constant ϵ which, in turn, increases the correlation of *P* and *F*. To counterbalance this self-enhancing dynamics, a second dynamic variable, i.e., β, is required. To make β increase, *F* and *x* have to be correlated which is the case once the oscillator has attained the externally applied frequency. This delay of the onsets of the growth processes of ϵ and β is crucial for the AFDC mechanism and cannot be realized by a single variable.

In this contribution, we focused on the Hopf oscillator and the Van der Pol oscillator for the detailed analyses of the regular AFO and the AFDC mechanism. It remains an interesting question for future research in how far the results obtained for these oscillators regarding the frequency-independent choice of parameters as well as regarding the quality measures of the adaptation process generalize to other types of nonlinear oscillators (Rayleigh, 1877; Duffing, 1918; Fitzhugh, 1961).

As the dynamics of the coupling strengths in the AFDC mechanism is solely correlation based, we showed that it is easy to implement the mechanism in neural networks. We demonstrated this by discussing the already earlier published neural time-discrete SO(2)-oscillator with AFDC mechanism (Nachstedt et al., 2012). This special realization of the AFDC mechanism has already been successfully applied in different robotic applications including self-organized control of a snake-like robot (Nachstedt et al., 2013), adaptive control of a robot leg with compliant tarsus (Canio et al., 2016b), and bipedal locomotion with robustness against global loss of sensory feedback (Canio et al., 2016a). In contrast to the neural implementation, the new general formulation of the AFDC mechanism makes it possible to apply the mechanism to all kinds of existing applications of regular AFOs (Buchli et al., 2005). Additionally, it allows the usage of adaptive oscillators in completely new scenarios where, up to now, regular AFOs could not provide sufficiently fast as well as precise adaptation.

## 4. Materials and methods

### 4.1. Hopf oscillator

The regular Hopf oscillator with the state variables *x* and *y* is given by the following system of dynamical equations:

(9) $$\begin{array}{l} \dot{x}(t)=\left(\mu -r{\left(t\right)}^{2}\right)x(t)-\theta y(t)\\ \dot{y}(t)=\left(\mu -r{\left(t\right)}^{2}\right)y(t)+\theta x(t) \end{array}$$

with $r\left(t\right)=\sqrt{x{\left(t\right)}^{2}+y{\left(t\right)}^{2}}$. The variable μ > 0 determines the amplitude of the oscillations. Without an external signal (*F*(*t*) = 0), this system possesses an asymptotically stable and harmonic limit cycle with an angular frequency of exactly θ.

#### 4.1.1. Adaptive frequency Hopf oscillator

The Hopf oscillator can be turned into an adaptive frequency oscillator by coupling an external signal *F* to the system and introducing the dynamics described by Equation (2) to the parameter θ. The complete system is given by

(10) $$\begin{array}{l} \dot{x}(t)=\left(\mu -r{\left(t\right)}^{2}\right)x(t)-\theta (t)y(t)+\epsilon F(t)\\ \dot{y}(t)=\left(\mu -r{\left(t\right)}^{2}\right)y(t)+\theta (t)x(t)\\ \dot{\theta }(t)=-\eta F(t)\frac{y(t)}{\sqrt{x{\left(t\right)}^{2}+y{\left(t\right)}^{2}}}. \end{array}$$

#### 4.1.2. Hopf oscillator with fast dynamical coupling

The Hopf oscillator equipped with the AFDC mechanism is given by the following system of differential equations:

(11) $$\begin{array}{l} \dot{x}(t)=\left(\mu -r{\left(t\right)}^{2}\right)\;x(t)-\theta (t)y(t)+P(t)\\ \dot{y}(t)=\left(\mu -r{\left(t\right)}^{2}\right)\;y(t)+\theta (t)x(t)\\ \tau \dot{\beta }(t)=\beta_{0}-\beta (t)+\kappa P(t)x(t)\\ \tau \dot{\epsilon }(t)=\epsilon_{0}-\epsilon (t)+\kappa F(t)P(t)\\ \dot{\theta }(t)=-\eta P(t)\frac{y(t)}{\sqrt{x{\left(t\right)}^{2}+y{\left(t\right)}^{2}}} \end{array}$$

with *P*(*t*) = ϵ(*t*)*F*(*t*) − β(*t*)*x*(*t*).

### 4.2. Van der Pol oscillator

The Van der Pol oscillator with the state variables *x* and *y* is defined as follows:

(12) $$\begin{array}{l} \dot{x}(t)=y(t)\\ \dot{y}(t)=\mu \left(1-x{\left(t\right)}^{2}\right)y(t)-\theta^{2}x(t). \end{array}$$

The parameter μ > 0 determines the “degree of nonlinearity” of the system. For μ = 0, the system is harmonic. The intrinsic frequency *f* depends in a nonlinear and non-trivial way on the parameter θ. We use a Fourier transform in conjunction with a sequence of nested intervals to determine the values of θ corresponding to a given frequency *f*.

#### 4.2.1. Adaptive frequency Van der Pol oscillator

The adaptive frequency formulation of the Van der Pol Oscillator coupled to a time-dependent external signal *F*(*t*) requires a positive sign in Equation (2):

(13) $$\begin{array}{l} \dot{x}(t)=y(t)+\epsilon F(t)\\ \dot{y}(t)=\mu (1-x{\left(t\right)}^{2})y(t)-\theta {\left(t\right)}^{2}x\\ \dot{\theta }(t)=+\eta F(t)\frac{y(t)}{\sqrt{x{\left(t\right)}^{2}+y{\left(t\right)}^{2}}}. \end{array}$$

#### 4.2.2. Van der Pol oscillator with fast dynamical coupling

Applying the AFDC mechanism to the Van der Pol oscillator is described by the following system of differential equations:

(14) $$\begin{array}{l} \dot{x}(t)=y(t)+P(t)\\ \dot{y}(t)=\mu (1-x{\left(t\right)}^{2})y(t)-\theta {\left(t\right)}^{2}x\\ \tau \dot{\beta }(t)=\beta_{0}-\beta (t)+\kappa P(t)x(t)\\ \tau \dot{\epsilon }(t)=\epsilon_{0}-\epsilon (t)+\kappa F(t)P(t)\\ \dot{\theta }(t)=+\eta P(t)\frac{y(t)}{\sqrt{x{\left(t\right)}^{2}+y{\left(t\right)}^{2}}} \end{array}$$

with *P*(*t*) = ϵ(*t*)*F*(*t*) − β(*t*)*x*(*t*).

### 4.3. Neural SO(2)-oscillator

We use standard additive time-discrete neurons *H*_*i*_, *i* ∈ {0, …, *N* − 1}, where *N* is the number of neurons in the network. The activation *a*_*i*_ of neuron *H*_*i*_ at time *t* + 1 is given by the sum of incoming presynaptic neural firing rates *o*_*j*_ weighted by the synaptic weights *w*_*ij*_ at time *t*:

(15) $$a_{i}(t+1)=\sum_{j\;=\;0}^{N-1}w_{ij}(t)o_{j}(t),\;\;\;i=0,\ldots ,N-1.$$

The activation *a*_*i*_ of neuron *H*_*i*_ is transformed into its firing rate *o*_*i*_ by a sigmoidal transfer function:

(16) $$o_{i}(t)=\text{tanh }\left(a_{i}(t)\right).$$

The pure SO(2)-network consists of *N* = 2 fully connected neurons *H*_0_ and *H*_1_. The synaptic weight matrix is chosen according to

(17) $$\left(\begin{array}{cc} w_{00}(t) & w_{01}(t)\\ w_{10}(t) & w_{11}(t) \end{array}\right)=\alpha \;\cdot \;\left(\begin{array}{cc} \text{cos}\varphi (t) & \text{sin}\varphi (t)\\ -\text{sin}\varphi (t) & \text{cos}\varphi (t) \end{array}\right)$$

with 0 < φ(*t*) < π the frequency determining parameter. The factor α determines the amplitude as well as the nonlinearity of the oscillations. We use α = 1.01 to obtain very harmonic oscillations and an approximately linear relationship between φ and the intrinsic frequency of the oscillator.

#### 4.3.1. SO(2)-oscillator with fast dynamical coupling

In order to equip the neural SO(2)-oscillator with the AFDC mechanism, an additional neuron *H*_2_ is introduced. The external signal *F*(*t*) is fed into the neuron *H*_2_ via a synapse *w*_2*F*_. The neuron *H*_2_ calculates the filtered version of the external signal and receives signals via the synapses *w*_20_ (= β) and *w*_2*F*_ (= ϵ) governed by the following plasticity rules:

(18) $$\begin{array}{l} w_{20}(t+1)=w_{20}(t)+(\beta_{0}-w_{20}(t)-\kappa o_{2}(t)o_{0}(t))/\tau \\ w_{2F}(t+1)=w_{2F}(t)+(\epsilon_{0}-w_{2F}(t)-\kappa F(t)o_{2}(t))/\tau . \end{array}$$

In accordance with our earlier publication (Nachstedt et al., 2012), we simplify the frequency adaptation rule of the AFDC mechanism and reformulate it in terms of the signals arriving at neuron *H*_0_:

(19) $$\varphi (t+1)=\phi (t)+\eta w_{02}(t)o_{2}(t)w_{01}(t)o_{1}(t).$$

For the example adaptation process (Figure 10B), we use α = 1.01, η = 1, κ = 100, τ = 100, β_0_ = 0 and ϵ_0_ = 0.01.

### 4.4. Mathematical pendulum

The angular displacement λ of a mathematical pendulum with length *l* and mass *m* is described by the following differential equation:

(20) $$\ddot{\lambda }=-\frac{g}{l}\text{sin}\lambda -\frac{D}{ml^{2}}\dot{\lambda }+\frac{M}{ml^{2}}$$

with the gravitational acceleration *g*, the external torque *M* evoked on the system and the damping constant *D*. The resonant frequency *f*_res_ of the undamped (*D* = 0) and undriven (*M* = 0) mathematical pendulum is given by Ochs (2011):

(21) $$f_{\text{res}}=\frac{\omega_{0}}{4K(k)}$$

with

(22) $$k=\frac{{\dot{\lambda }}^{2}+4\omega_{0}^{2}{\left(\text{sin}\frac{\lambda }{2}\right)}^{2}}{4\omega_{0}^{2}}$$

and $\omega_{0}=\sqrt{\frac{g}{l}}$. *K*(*k*) is the complete elliptic integral of the first kind. In Equation (22), the current values of the angular displacement λ and the angular velocity $\dot{\lambda }$ are used to obtain the current total energy of the system. For our simulations, we use *g* = 9.81 mm ^−2^ and *D* = 0.005 kg m^2^ s^−1^.

### 4.5. Numerical integration

The integrations of the different differential systems are carried out using the odeint method of the scipy python package (Jones et al., 2001). This methods relies on the LSODA algorithm (Brown and Hindmarsh, 1989) from the FORTRAN library odepack (Hindmarsh, 1983). The LSODA algorithm utilizes an adaptive step size.

### 4.6. Frequency and parameter scans

For the frequency scans performed for the adaptive Hopf oscillator (Figures 4, 5) and the adaptive Van der Pol oscillator as well as for the respective oscillators with AFDC mechanism (Figures 7, 8), we sample the frequency space in the range 0.1 ≤ *f*_0_, *f*_ext_ ≤ 10.0. We consider 21 sample values uniformly spaced on a logarithmic axis of *f*_0_ and *f*_ext_ and investigate the behavior of the oscillators for all possible 21^2^ (*f*_0_, *f*_ext_)-pairs. For every frequency pair, in the case of the regular adaptive oscillators, we sample 21 parameter values again uniformly spaced on a logarithmic axes of each ϵ and η in the range 0.01 ≤ ϵ, η ≤ 100. Therefore, we investigate a total of 21^4^ (*f*_0_, *f*_ext_, ϵ, η)-configurations for each regular adaptive oscillator. In the case of the oscillators with AFDC mechanism, the parameters are investigated in the ranges 0.01 ≤ η, τ ≤ 100 and 1 ≤ κ ≤ 1, 000 again with 21 samples in every parameter dimension yielding a total of 21^5^ sampled (*f*_0_, *f*_ext_, η, κ, τ)-configurations each.

The best sampled parameter values of the frequency space averaged combined quality measure 〈*Q*〉 are ϵ ≈ 15.85 and η ≈ 15.85 for the regular adaptive Hopf oscillator and η ≈ 1.58, κ ≈ 398.11 and τ ≈ 3.98 for the Hopf oscillator with AFDC mechanism (Figure 9). For the regular adaptive Van der Pol oscillator, we find ϵ ≈ 0.0158 and η = 1.0 to perform best while η ≈ 0.158, κ = 100 and τ ≈ 1.585 yield the best result for the Van der Pol oscillator with AFDC mechanism.

## Author contributions

The AFDC mechanism was developed by TN and PM. TN and CT planned the presented analyses and experiments. Implementation and analysis of the data was done by TN. TN, CT, and PM wrote and reviewed the manuscript.

## Funding

The research leading to these results has received funding from the Federal Ministry of Education and Research (BMBF) Germany to the Göttingen Bernstein Center for Computational Neuroscience under grant numbers 01GQ1005A [TN, PM] and 01GQ1005B [CT] and from the International Max Planck Research School for Physics of Biological and Complex Systems (IMPRS-PBCS) by stipends of the country of Lower Saxony with funds from the initiative Niedersächsisches Vorab and of the University of Göttingen [TN].

### Conflict of interest statement

The authors declare that the research was conducted in the absence of any commercial or financial relationships that could be construed as a potential conflict of interest.
